# Effects of exercise-related interventions on blood pressure and related variables among individuals with hypertension: a systematic review and meta-analysis

**DOI:** 10.3389/fcvm.2026.1908252

**Published:** 2026-10-13

**Authors:** Babangida Shehu Bappah, Umar Muhammad Bello, Aisha Shittu, Lawan Umar, Suleiman Mohammed, Buhari Abdullahi Tafida, Adamu Usman Gamawa, Ismail Idris, Zainab Umar Dahiru, Asmau Ahmad Tijjani, Dauda Salihu, Sabiu Hamza Muhammad, Jibril Mohammed Nuhu, Isah Usman Lawal, Jibril Mohammed

**Affiliations:** 1 Physiotherapy Department, Federal University of Health Sciences Azare, Azare, Nigeria; 2 Department of Allied Health Professions, School of Health and Life Sciences, Glasgow Caledonian University, Glasgow, United Kingdom; 3 Department of Physiotherapy, Faculty of Allied Health Sciences, Bayero University, Kano, Nigeria; 4 Department of Community, Psychiatric and Mental Health Nursing, College of Nursing, Qassim University, Buraydah, Saudi Arabia; 5 Department of Medicine, Federal University Dutse, Dutse, Nigeria

**Keywords:** aerobic exercise, blood pressure, exercise training, hypertension, meta-analysis, resistance training, systematic review

## Abstract

**Background:**

Hypertension is a leading contributor to global cardiovascular disease burden and premature mortality. Although pharmacological treatment is effective, long-term Blood Pressure (BP) control remains suboptimal for many patients, highlighting the importance of non-pharmacological strategies such as physical exercise. This systematic review and meta-analysis aimed to evaluate the effectiveness of exercise training interventions for improving BP outcomes among adults with hypertension. While a limited number of included randomized controlled trials involved participants with prehypertension, the review primary focus was hypertension management.

**Methods:**

A systematic literature search was conducted across five electronic databases (Scopus, AMED, MEDLINE, PUBMED, and CINAHL) to identify relevant clinical trials. A small number of trials involving participants with prehypertension or stage 1 hypertension were retained where they met the predefined eligibility criteria and contributed to the overall evidence regarding elevated BP management. Methodological quality of the included studies was assessed using the PEDro scale. Data were synthesized using random-effects meta-analysis, and pooled effects were expressed as standardized mean differences (SMDs) with 95% confidence intervals (CIs). Statistical heterogeneity was evaluated using the I^2^ statistic. Meta-analyses were conducted using Review Manager 5. Structured Narrative synthesis was performed where meta-analysis was not feasible.

**Results:**

A total of 26 studies that recruited 1,576 participants met the inclusion criteria and were included in the review. Majority of the studies were of moderate-to-good methodological quality (∼70%). Aerobic training consistently produced statistically significant and clinically meaningful reductions in both SBP (SMD: −0.97; 95% CI −1.46 to −0.47; *p* = 0.0001) and DBP (SMD: −1.55; 95% CI −2.19 to −0.90; *p* < 0.00001). Combined aerobic and resistance training also resulted in significant reductions in SBP (SMD: −1.11; 95% CI: −2.11 to −0.11; *p* = 0.03) and DBP (SMD: −1.25; 95% CI: −2.24 to −0.27; *p* = 0.01), suggesting potential additive or complementary effects of multimodal exercise interventions. Resistance training alone demonstrated a significant and large reduction in DBP but showed inconsistent and non-significant effects on SBP. In addition to blood pressure reduction, several studies reported improvements in secondary outcomes, including cardiorespiratory fitness, heart rate variability, body composition, inflammatory markers, physical performance, and quality of life. However, substantial between-study heterogeneity was observed across most pooled analyses, warranting cautious interpretation of the findings.

**Conclusion:**

The available evidence suggests that aerobic exercise, either alone or combined with resistance training, is associated with some beneficial reductions in systolic and diastolic blood pressure among individuals with hypertension.

## 1 Background

Hypertension is defined as a persistent elevation in systolic blood pressure (SBP) ≥ 140 mmHg and or diastolic blood pressure (DBP) ≥ 90 mmHg (1, 2). It is a leading risk factor for cardiovascular diseases (CVDs), and kidney failure (3, 4). Hypertension is a leading contributor to the global burden of disease, with its impact quantified through key metrics such as disability-adjusted life years (DALYs), years of life lost (YLLs), and years lived with disability (YLDs) (5). According to the World Health Organization (WHO), approximately 1.13 billion people worldwide suffer from hypertension (6). An estimated 40% of adults over the age of 25 years have been reported to be affected by hypertension (4, 7). Despite advancements in pharmacological management of hypertension over the years, it remains a major public health challenge in many parts of the world (8, 9).

Lifestyle modifications including physical activity are increasingly recognized as essential components of hypertension management, both in terms of primary prevention and as an adjunct to pharmacotherapy among those already diagnosed (10, 11). Presently, the role of physical activity in the prevention and management of hypertension has been documented in several studies. The current evidence seem to suggest that participating in regular exercise training can lower Blood Pressure (BP) (11–13), improve vascular health, and enhance overall cardiovascular fitness among other benefits (14, 15). Similarly, various types of physical activity programs, have shown promise in reducing both systolic and diastolic BP (16–18).

The use of non-pharmacological treatment options for managing hypertension particularly in cases where medication alone may not be sufficient or among individuals seeking to reduce their reliance on hypertensive medications is gaining attention (19, 20). Some of these non-pharmacological strategies include dietary changes, such as adopting the Dietary Approaches to Stop Hypertension (DASH) diet, regular physical activity (21–23), reducing body weight (24), reducing stress through mindfulness, relaxation techniques, getting adequate sleep (25), and quitting smoking. These non-pharmacological interventions, either alone or in combination with pharmacotherapy, offer effective and sustainable methods for managing hypertension, particularly when implemented as part of a holistic, patient-centered approach (8, 26–28).

Exercise lowers blood pressure through multiple complementary physiological mechanisms (11). Aerobic exercise improves endothelial function and nitric oxide bioavailability, reduces sympathetic nervous system activity, enhances arterial compliance and decreases peripheral vascular resistance (13). Resistance training may improve autonomic regulation, vascular function and skeletal muscle metabolism, while combined exercise may produce additive cardiovascular adaptations through improvements in both cardiorespiratory fitness and muscular strength (11). These mechanisms provide a biological rationale for evaluating the relative effectiveness of different exercise modalities.

Despite the promising results of the effects of exercise in managing hypertension and the well-established therapeutic mechanisms, there is considerable variability in the findings across existing studies especially in terms of prescription of exercise training component of non-pharmacological interventions (29, 30). Consequently, there is a need to summarize and quantify the evidence to support the effectiveness of exercise training interventions for managing hypertension.

Although exercise training and blood pressure reduction have been extensively investigated, important differences remain in the scope, populations, exercise modalities and outcomes addressed by previous reviews (19, 20, 31–33). Pescatello et al. synthesized evidence across adults with normal blood pressure, prehypertension and hypertension, providing broad evidence that physical activity reduces blood pressure across the elevated blood pressure spectrum (33). More recently, Edwards et al. conducted a large pairwise and network meta-analysis involving 270 randomized controlled trials and 15,827 participants and demonstrated significant reductions in resting systolic and diastolic blood pressure following aerobic, dynamic resistance, combined, high-intensity interval and isometric exercise (34). Against this background, the contribution of the present review is not to establish that exercise lowers BP, but to provide a focused synthesis of randomized trials identified through 2024, with separate quantitative analyses of aerobic, resistance and combined exercise interventions among predominantly hypertensive populations. The review also integrates quantitative BP outcomes with reported functional and cardiovascular outcomes and highlights areas in which the available modality-specific evidence remains limited or heterogeneous. This focused approach may assist clinicians and researchers in interpreting the current evidence while avoiding assumptions of superiority where the evidence remains insufficient.

## 2 Methods

### 2.1 Study design

The review followed the *Preferred Reporting Items for Systematic Reviews and Meta-Analyses* (PRISMA) guidelines (35). The protocol was registered on PROSPERO (CRD42024571261). All steps, including literature search, study selection, data extraction, and analysis, were conducted by multiple researchers to ensure the robustness of the review process.

### 2.2 Information sources

A comprehensive and systematic literature search was conducted in 2024. The search strategy was developed using the Participants, Intervention, Comparison, Outcome and Study design (PICOS) framework, which identifies the following elements: Hypertension (P), exercise training-related interventions (I), No specific control (C), Blood pressure (O) and Randomized Control Trials (S) respectively. Electronic databases were searched including Scopus, AMED, MEDLINE, PUBMED, and CINAHL. The search was restricted to randomized controlled trials (RCTs) published between January 2004 (after the seventh report of the joint national committee on prevention, detection, evaluation, and treatment of high blood pressure guidelines in 2003 (2) and 2024 because this period reflects contemporary hypertension diagnosis and management following publication of JNC7 and coincides with the widespread adoption of standardized exercise prescription protocols in hypertension research.

The search was conducted using a combination of relevant keywords and Medical Subject Headings (MeSH) terms associated with the PICOS elements. (See Table 1 for the detailed PICO framework). Generated studies through the electronic searches were managed using Rayyan (36). The complete database-specific search strategies, including MeSH terms, keywords, Boolean operators, database-specific syntax, filters, and publication limits, are presented in Table 2 to facilitate reproducibility.

**Table 1:** PICO and keywords utilized in the review.

Population: known hypertensive patient	Intervention: physical activity related intervention	Control: non-physical activity intervention	Outcome: decreased blood pressure	Study design
*“hypertensive patients” “hypertension” “HTN” “high blood pressure”*, *increased BP*, *high systolic BP*, and *high diastolic BP*	*physical activity*, *PA*, *exercise*, *aerobic exercise*, *exercise training*, *brisk walking*, *jogging*, *treadmill exercise*, and *fitness training*.	*Control*	*decreased systolic pressure*, *decreased diastolic pressure*, *decreased SBP*, *decreased DBP*, and *blood pressure control*	*“RCT” ‘’Clinical trials’’*

**Table 2:** Complete search strategy used for the electronic database search.

Database	Search strategy
PubMed	[“Hypertension"(MeSH) OR hypertension OR “high blood pressure” OR “elevated blood pressure”] AND [“Exercise"(MeSH) OR exercise OR “physical activity” OR “exercise training” OR aerobic OR walking OR cycling OR swimming OR “resistance training” OR “strength training” OR “combined exercise” OR “isometric exercise”] AND [“Blood Pressure"(MeSH) OR “blood pressure” OR systolic OR diastolic] AND [“Randomized Controlled Trial"(Publication Type) OR randomized OR randomised OR RCT].**Filters:** Humans, Adults (≥18 years), English language, January 2004–December 2024.
MEDLINE (via EBSCOhost)	(MH “Hypertension+” OR hypertension OR “high blood pressure”) AND (MH “Exercise+” OR MH “Motor Activity+” OR exercise OR “physical activity” OR aerobic OR walking OR cycling OR swimming OR “resistance training” OR “strength training” OR “combined exercise” OR “isometric exercise”) AND (MH “Blood Pressure+” OR “blood pressure” OR systolic OR diastolic) AND (MH “Randomized Controlled Trials+” OR randomized OR randomised OR clinical trial). **Limits:** Human, Adult, English, 2004–2024
CINAHL	(MH “Hypertension+”) OR hypertension OR “high blood pressure” AND (MH “Exercise+”) OR (MH “Exercise Therapy+”) OR “physical activity” OR aerobic OR walking OR cycling OR swimming OR “resistance training” OR “combined exercise” OR “isometric training” AND (MH “Blood Pressure+”) OR systolic OR diastolic AND randomized controlled trial OR randomized OR RCT. **Limiters:** English language; Adults; Human; Published January 2004–December 2024
Scopus	TITLE-ABS-KEY (hypertension OR “high blood pressure”) AND TITLE-ABS-KEY (“physical activity” OR exercise OR “exercise training” OR aerobic OR walking OR cycling OR swimming OR “resistance training” OR “strength training” OR “combined exercise” OR “isometric exercise”) AND TITLE-ABS-KEY (“blood pressure” OR systolic OR diastolic) AND TITLE-ABS-KEY (randomized OR randomised OR “randomized controlled trial” OR RCT). **Limits:** English language; Articles; 2004–2024
AMED	Hypertension OR “high blood pressure”) AND (Exercise OR “physical activity” OR aerobic OR walking OR cycling OR swimming OR “resistance training” OR “combined exercise” OR “isometric exercise”) AND (“blood pressure” OR systolic OR diastolic) AND (randomized OR randomised OR “clinical trial”). **Limits:** Human adults; English language; January 2004–December 2024.

MeSH, medical subject headings; MH, CINAHL subject heading; RCT, randomized controlled trial; TITLE-ABS-KEY, scopus search field (title, abstract, keywords).

### 2.3 Eligibility criteria

Randomized controlled trials involving adults (≥18 years) with established hypertension were eligible for inclusion. We also retained a small number of randomized trials that recruited participants with prehypertension or stage 1 hypertension when these studies evaluated structured exercise interventions specifically targeting elevated blood pressure and otherwise fulfilled all eligibility criteria. This decision was made because some exercise trials recruit participants across the elevated blood pressure spectrum, and excluding such studies solely on the basis of BP category could result in loss of potentially relevant randomized evidence concerning early blood pressure management. However, these populations were not considered interchangeable with established hypertension. Therefore, the primary interpretation of this review remains focused on adults with hypertension, while the inclusion of prehypertensive or stage 1 hypertensive participants is considered a potential source of clinical heterogeneity. Studies focusing exclusively on secondary hypertension and studies that did not report quantitative BP outcomes were excluded. Conference abstracts, proceedings and review articles were also excluded.

### 2.4 Selection process

Prior to formal screening, reviewers independently screened a sample of retrieved records to ensure consistent interpretation of the eligibility criteria. Screening and full-text assessment were conducted independently by two reviewers, with disagreements resolved through discussion and consultation with a third reviewer where necessary. The initial screening of tittle and abstract was performed by two researchers who were initially blinded to each other's assessment (BSB and LU) on the Rayyan software (36). The abstracts were screened to ensure that the study included exercise training, physical activity interventions and hypertension management. Areas of conflict were resolved by consulting a third (JM). The available articles were further downloaded and subjected to full text screening. The full-text screening was conducted by BSB and AUG. Studies were excluded if they did not meet the inclusion criteria.

### 2.5 Methodological quality assessment

The methodological quality of included studies was assessed using the PEDro scale. PEDro was selected because it is widely validated and extensively used in physiotherapy and exercise intervention trials, providing a standardized assessment of methodological quality specifically designed for rehabilitation research. This scale assesses 11 criteria, such as randomization, allocation concealment, and blinding, to determine the methodological quality in RCTs. Each study was assigned a score based on the PEDro scale, and the average score for each article was calculated and recorded by two independent assessors (BSB and DS). Any conflict in the methodological quality assessment was resolved by consensus among the reviewers. Studies were categorized as having *low*, *moderate*, or *high* methodological quality based on their PEDro scores (29).

### 2.6 Data extraction

Data extraction was performed by one of the researchers (BSB) and later verified by a second researcher (AUG) using a pre-designed Excel sheet. An improvised data extraction sheet was used to record the extracted data.

### 2.7 Data synthesis

A narrative and quantitative data synthesis was carried out to summarise the effects of exercise-based interventions on SBP and DBP among individuals with hypertension. Standardized mean difference (SMD) was used as an effect size in the meta-analyses because the included studies reported blood pressure outcomes using different statistical approaches, including post-intervention values, change-from-baseline scores, and adjusted between-group differences, with considerable variation in study populations, intervention characteristics, and outcome variability. Although blood pressure is measured in millimetres of mercury, SMDs enabled standardized synthesis across heterogeneous reporting formats while maximizing inclusion of eligible randomized controlled trials. To enhance clinical interpretation, observed reductions in systolic and diastolic BP (mmHg) are also presented narratively. Given variations in exercise type, intensity, duration, population characteristics, and outcome reporting across the included studies, a random-effects model was applied. Where pooling was not feasible due limited number of homogeneous, findings were synthesised narratively. Studies were grouped according to exercise modality, and outcomes were synthesised separately for SBP and DBP. Secondary cardiovascular and functional outcomes (e.g., heart rate, VO₂max, quality of life, inflammatory markers, and body composition) were descriptively summarised to support interpretation of primary outcomes. The number of studies evaluating an intervention, the methodological quality of the studies and the consistency of the available evidence was used to generate a 5-level of evidence (37) using the modified Bakker scale (38) as presented in Table 3.

**Table 3:** Modified bakker scale.

Level of evidence	Criteria
Strong evidence	Consistent findings in 2 or more good quality studies, or 1 good quality and at least 2 fair quality studies.
Moderate evidence	Consistent findings in 1 good quality study plus 1 fair quality study, or 2 fair quality studies.
Limited evidence	Only 1 fair or poor quality study is available.
Conflicting evidence	Inconsistent findings in the available studies.
Weak evidence	Consistent findings in 1 fair quality study plus 1 or more poor, or 2 or more poor, or only 1 good quality study.

Consistent is when at least 75% of the available studies reported the same conclusion.

## 3 Results

A total of 6254 articles were retrieved and imported to Rayyan. After the initial import, 706 duplicate entries were automatically identified and removed. 5,548 articles were assessed for eligibility. 150 titles were selected for abstract screening. Subsequently, 56 full-text articles were retrieved for full text screening. Following this screening, 26 articles met the inclusion criteria and were included in the final analysis (Figure 1).

**Figure 1:**
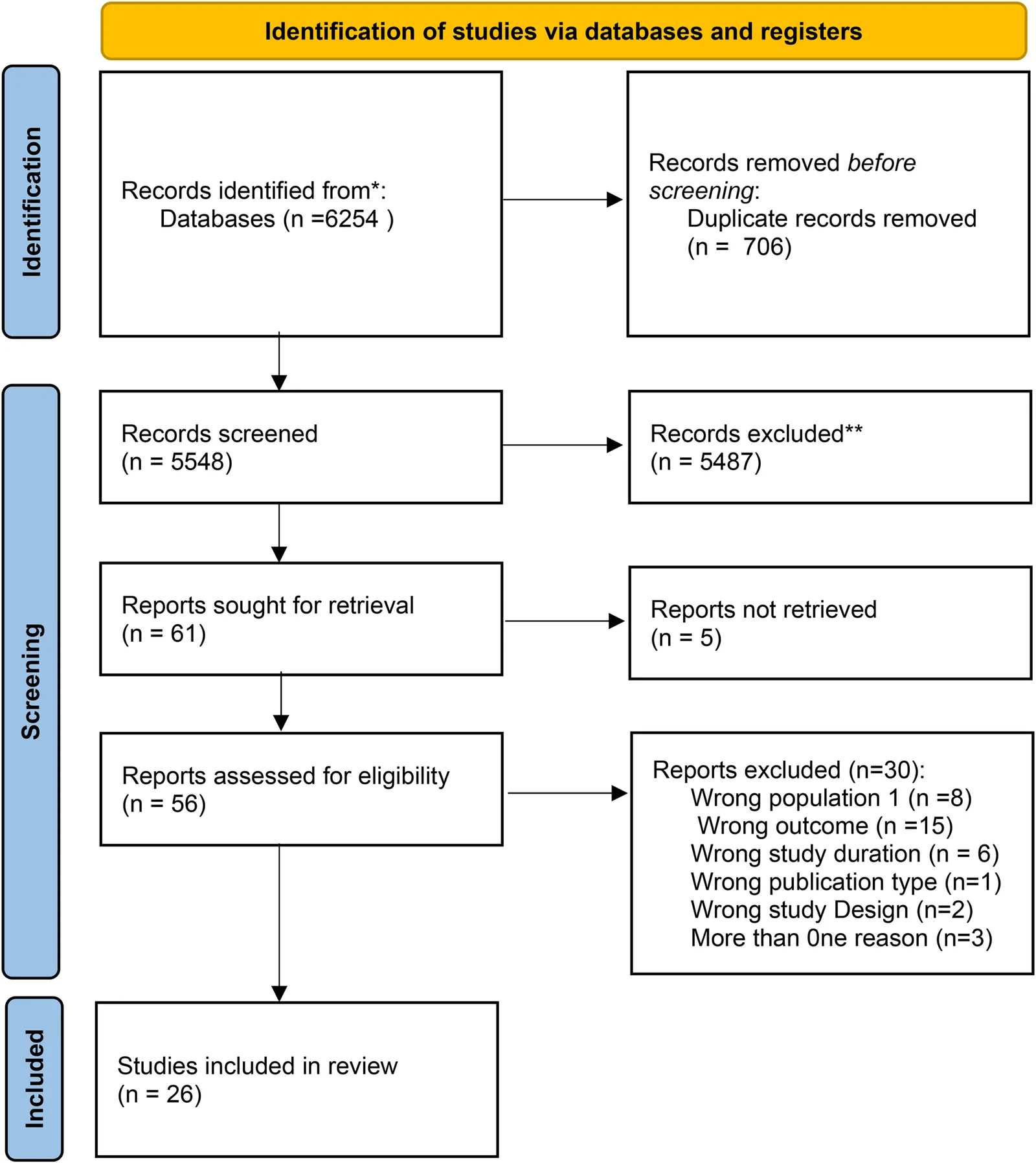
PRISMA flow chat for systematic review.

### 3.1 Characteristics of included studies

A total of 26 studies met the inclusion criteria and were included in the systematic review, with most studies also contributing to the quantitative meta-analysis (Table 4). Most included studies enrolled adults with established hypertension. A small number of studies included participants with prehypertension or stage 1 hypertension; however, these represented only a minority of the overall evidence base. Consequently, the findings of this review primarily reflect the effects of exercise interventions among adults with hypertension. The included studies were published between 2008 and 2024 and involved a total sample size exceeding 1,576 participants across diverse populations including elderly hypertensive patients, sedentary adults, individuals with resistant hypertension, and patients with stage 1 hypertension. Intervention durations ranged from acute exercise sessions to structured training programs lasting 10–12 weeks or longer. Several studies also assessed secondary cardiovascular outcomes, including heart rate (HR), cardiorespiratory fitness (VO₂max), heart rate variability. inflammatory biomarkers, quality of life, physical performance and body composition.

**Table 4:** Included studies.

SNO	Author	Tittle	N	Intervention	BP Outcome (mmHg) MD(Pre-Post Intervention)	Control	Secondary outcome
1.	Alemayehu and Teferi	Effectiveness of Aerobic, Resistance, and Combined Training for Hypertensive Patients: A Randomized Controlled Trial		Aerobic Training Resistance training Combined training	SBP -12.50 (154.11- 141.61) DBP-9.75 (92.96-82.21)		HR VO2Max
2.	Caminiti et al.	Effects of 12 weeks of aerobic versus combined aerobic plus resistance exercise training on short-term blood pressure variability in patients with hypertension		Aerobic Combined aerobic plus resistance exercise training	SBP -4.9 (119.0 ± 13.3- 114.1 ± 12.7), -4.1 (118.8 ± 12.9- 114.7 ± 12.5) DBP -5.1 (71.7 ± 9.8- 65.6 ± 8.5), -3.2 (70.9 ± 10.0- 67.7.10.1)		HR
3.	Pathan et al.	Effect of slow breathing exercise and progressive muscle relaxation technique in the individual with essential hypertension. A randomized controlled trial	64 36 Male 28 Female	Slow breathing exercise Progressive muscle relaxation	SBP -6.09 DBP -4.00		Perceived Stress
4.	Palmeira et al.	Effects of isometric handgrip training on blood pressure among hypertensive patients seen within public primary healthcare: a randomized controlled trial	63 HTN pt	Resistance Exercise (Isometric Handgrip Exercise)	SBP -8.0 (129 ± 4-121 ± 3) DBP-4 (83 ± 3-79 ± 2)	SBP 0 (126 ± 3-126 ± 4) DBP-4 (81 ± 3-77 ± 3)	
5.	Lima et al.	Combined aerobic and resistance training: are there additional benefits for older hypertensive adults?	44	Aerobic training, Resistance training	SBP +5.0 (130.3-135.3) DBP +1.8 (75.5-77.3)	SBP +5.0 (130.3-135.3) DBP +4.5 (123.6-128.1)	Body composition
6.	Bocalini et al.	Post-exercise hypotension and heart rate variability response after water- and land ergometry exercise in hypertensive patients	45 Women	Aerobic Exercise	SBP -3 (153 ± 2-150 ± 2) DBP-3 (91 ± 1-88 ± 1)		Body Composition Heart rate variability
7.	Ciolac et al.	Acute aerobic exercise reduces 24-h ambulatory blood pressure levels in long-term-treated hypertensive patients	50	Aerobic exercise	SBP -7.1 (134.6-127.5) DBP-10 (91.5-81.5)		
8.	Javidi et al.	Effect of Lower- versus Higher-Intensity Isometric Handgrip Training in Adults with Hypertension: A Randomized Controlled Trial	39 Male	Isometric handgrip	SBP -15.5 DBP-5.0		Heart rate variability Serum Markers of Inflammation and Oxidative Stress
9.	Qin et al.	Effects of the Otago Exercise Program in older hypertensive patients with pre-frailty	75	Aerobic Exercise Resistance exercise	SBP -5.2 (141.2 ± 19.5-136.0 ± 11.5) DBP –9 (89.1 ± 14.9-80.1 ± 10.1)	SBP -3.7 (145.3 ± 18.3-141.6 ± 11.3) DBP -3.3 (87.0 ± 12.7-83.7 ± 11.3)	Frailty (Fatigue, Resistance, Ambulation, Illness, and Loss of Weight)
10.	Venturelli et al.	Effects of endurance, circuit, and relaxing training on cardiovascular risk factors in hypertensive elderly patients	40	Aerobic Exercise	SBP -19.4 DBP-8.0		Body Composition QOL
11.	Lamina	Effects of Continuous and Interval Training Programs in the Management of Hypertension: A Randomized Controlled Trial	357	Continuous training Interval training	SBP -15.7 (166.05 ± 14.10-150.35 ± 16.67)-14.43 (168.81 ± 12.73-154.38 ± 12.63) DBP -1.82 (96.80 ± 3.38-94.98 ± 5.04)-6.16 (100.63 ± 7.06-94.44 ± 8.77)	SBP +2.86 (160.87 ± 12.23-163.47 ± 14.88) DBP -1.07 (97.17 ± 1.43-96.10 ± 2.67)	VO2Max
12.	Farahani et al.	The Effects of a 10-Week Water Aerobic Exercise on the Resting Blood Pressure in Patients with Essential Hypertension	40	Water Aerobic Exercise	SBP -12.67 (154.17-141.50) DBP-0.04 (96.25-96.21)	SBP -1.78 (146.60-144.82) DBP +0.18 (90.89-91.07)	
13.	Yakasai et al.	Moderate intensity endurance exercise: a beneficial intervention for relative cardiovascular parameters of primary and secondary hypertensive patients. Randomised controlled trial	69	Moderate intensity endurance exercise	SBP -9.91 (129.91 ± 9.22-120.00 ± 9.85) Sec -9.36 (133.93 ± 10.09-124.57 ± 9.97) DBP -10.49 (91.10 ± 9.70-80.61 ± 6.80) Sec -9.94 (90.30 ± 7.63-80.36 ± 60)	SBP -0.57 (134.23 ± 11.34-133.66 ± 11.86) Sec +1.32 (130.00 ± 9.40-131.32 ± 12.73) DBP +1.52 (91.60 ± 5.82-93.12 ± 5.30) Sec -0.02 (90.52 ± 6.60-90.50 ± 8.20)	HR
14.	Boeno et al.	Effect of aerobic and resistance exercise training on inflammation, endothelial function and ambulatory blood pressure in middle-aged hypertensive patients	42	Aerobic exercise Resistance exercise	SBP -5.6 (118.2 ± 9.4-112.7 ± 78) RG -3.2 (119.9 ± 9.8-116.7 ± 7.3) SBP -2.5 (70.2 ± 7.1-67.7 ± 8.3) RG -3.7 (74.0 ± 7.4-70.3 ± 4.7)	SBP +2.8 (125.9 ± 9.7-128.7 ± 8.3) DBP +1.0 (81.9 ± 11.3-82.9 ± 10.5)	Body composition Inflammatory markers
15.	Sarinukul et al.	The Effects of Stepping Exercise on Blood Pressure, Physical Performance, and Quality of Life in Female Older Adults with Stage 1 Hypertension: a Randomized Controlled Trial	34	Stepping exercise	SBP -15.3 (147.3 ± 4.3-132.0 ± 6.9) DBP -11.7 (79.0-67.3)	SBP (144.1 ± 5.6-145.1 ± 5.9) DBP (84.3-87.6)	QOL Physical Performance
16.	Punia et al.	Effects of walking in individuals with prehypertension and stage 1 hypertension in India: a randomized controlled trial	63 Sedentary adults	Walking	SBP -5.53 (144.23 ± 8.96-138.70 ± 9.27) DBP -4.46 (91.33 ± 4.49-87.06 4.57)	SBP +5.82 (146.88 ± 5.06-152.70 ± 4.08) DBP +2.71 (94.64 ± 3.63-97.35 ± 4.04)	MAP PR
17.	Kruk and Nowicki	Effect of the physical activity program on the treatment of resistant hypertension in primary care	53 Patients with primary HTN	PA program	SBP -10.0 (150 ± 24-140.24) DBP -5.6 (90 ± 14-84.4 ± 13)		
18.	Garrett et al.	The antihypertensive effects of aerobic versus isometric handgrip resistance exercise	27	Aerobic Ex Isometric handgrip resistance ex	SBP -4.8 DBP -3.1		
19.	Silva et al.	Effects of Isometric Biceps Exercise on Blood Pressure in Adults with Hypertension	12	Isometric bicep exercise			
20.	Dimeo et al.	Aerobic Exercise Reduces Blood Pressure in Resistant Hypertension	50	Treadmill according to interval-training pattern	SBP -5.40 (135.3 ± 15.2-129.9 ± 10.0 DBP -2.80 (75.4 ± 9.5-72.6 ± 9.7)	SBP +2.3 (128.7 ± 12.2-131.1 ± 12.3) DBP +0.9 (70.2 ± 9.1-71.2 ± 7.1)	Arterial compliance Cardiac index
21.	Ruangthai et al.	Combined exercise training improves blood pressure and antioxidant capacity in elderly individuals with hypertension	54 older men	Combined exercise training	SBP -10.60 (142.0 ± 12.2-131.4 ± 15.9) DBP -8.10 (81.7 ± 5.6-73.6 ± 9.3)	SBP +6.7 (140.6 ± 18.2- 147.3 ± 20) DBP +1.0 (82.5 ± 10.1- 81.5 ± 12.1)	
22.	Hua et al.	Effects of Low-Intensity Exercise Conditioning on Blood Pressure, Heart Rate, and Autonomic Modulation of Heart Rate in Men and Women with Hypertension	40	Low-Intensity Exercise Conditioning.	SBP -11.10 DBP -5.20		Heart Rate Autonomic Modulation of Heart Rate
23.	Ngomane et al.	Hypotensive Effect of Heated Water-based Exercise in Older Individuals with Hypertension	15 (6 Men)	Heated Water-based Exercise	SBP +18.0 (132 ± 15- 150 ± 20) DBP +5.0 (81 ± 10- 86 ± 14)	SBP +1 (130 ± 11- 131 ± 10) DBP +2 (81 ± 10- 83 ± 12)	
24.	Ruangthai et al.	Comparative effects of water- and land-based combined exercise training in hypertensive older adults					
25.	Goncalves et al.	Functional and physiological effects of a 12-week programme of resistance training in elderly hypertensive women			SBP –3.1 (126.0 ± 5.2-122.9 ± 4.4) DBP +1.0 (80.9 ± 3.3-81.9 ± 4.4)	SBP -2.1(137.0 ± 5.0-134.9 ± 4.5) DBP +0.4(88.3 ± 2.6-88.7 ± 2.7)	

**Table 5:** Quality ratings: methodological quality within studies

No	Author	Year	Eligibility	Randomized allocation	Concealed allocation	Similarity at baseline	blinding of participant	Blinding of therapist	Blinding of assessor	Dropout	Intention to treat	Group comparison	PMVD	Total Score (10)	Internal validity subscale score(8)	Statistical reporting Sub scale (2)	Interpretation
1	Ciolac	2008	Yes	Yes	No	No	No	No	No	Yes	No	Yes	Yes	4	3	2	Moderate
2	Hua	2009	Yes	No	No	Yes	No	No	No	Yes	No	Yes	Yes	4	2	2	Moderate
3	Farahani	2010	Yes	No	No	Yes	No	No	No	Yes	No	Yes	Yes	4	2	2	Moderate
4	Alsairafi	2010	No	No	No	Yes	No	No	No	Yes	No	Yes	Yes	4	2	2	Moderate
5	Lamina	2010	Yes	Yes	No	Yes	No	No	No	No	No	Yes	Yes	4	2	2	Moderate
6	Dimeo	2012	Yes	Yes	No	Yes	No	No	No	Yes	No	Yes	Yes	5	3	2	Moderate
7	Goncalves	2014	No	Yes	No	No	No	No	No	No	No	Yes	Yes	5	3	2	Moderate
8	Venturelli	2015	Yes	Yes	No	Yes	No	No	Yes	Yes	No	Yes	Yes	6	4	2	Good
9	Bocalini	2017	Yes	Yes	No	Yes	No	No	No	No	No	Yes	Yes	4	2	2	Moderate
10	Lima	2017	No	Yes	No	Yes	No	No	No	No	No	Yes	Yes	4	2	2	Moderate
11	Ash	2017	Yes	Yes	No	Yes	Yes	No	No	Yes	Yes	Yes	Yes	7	5	2	Good
12	Kruk	2018	Yes	No	No	No	No	No	No	Yes	Yes	Yes	Yes	4	2	2	Moderate
13	Ngomane	2018	Yes	Yes	No	Yes	No	No	Yes	Yes	Yes	Yes	Yes	7	5	2	Good
14	Ruangthai	2019	Yes	Yes	No	Yes	No	No	No	Yes	No	Yes	Yes	5	3	2	Moderate
15	Boeno	2020	Yes	Yes	No	Yes	No	No	Yes	Yes	No	Yes	Yes	6	2	2	Good
16	Punia	2020	Yes	Yes	No	Yes	Yes	Yes	No	Yes	No	Yes	Yes	7	5	2	Good
17	Yakasai	2020	Yes	Yes	No	Yes	No	No	Yes	Yes	No	Yes	Yes	6	4	2	Good
18	Caminiti	2021	No	Yes	No	Yes	No	No	No	Yes	No	Yes	Yes	5	2	2	Moderate
19	Silva	2021	Yes	Yes	No	Yes	No	No	No	No	No	Yes	Yes	4	2	2	Moderate
20	Palmeira	2021	Yes	Yes	No	Yes	No	Yes	Yes	No	No	Yes	Yes	6	4	2	Good
21	Decaux	2022	No	Yes	No	Yes	No	No	No	Yes	No	Yes	Yes	5	3	2	Moderate
22	Javidi	2022	Yes	Yes	No	Yes	No	No	Yes	Yes	Yes	Yes	Yes	7	5	2	Good
23	Qin	2022	Yes	Yes	No	Yes	No	No	No	No	No	Yes	Yes	4	2	2	Moderate
24	Pathan	2023	Yes	Yes	No	No	No	No	Yes	Yes	Yes	Yes	Yes	6	4	2	Good
25	Alemayehu	2023	Yes	Yes	No	Yes	No	No	No	Yes	No	Yes	Yes	5	3	2	Moderate
26	Sarink	2023	Yes	Yes	Yes	Yes	No	No	No	Yes	No	Yes	Yes	6	4	2	Good

Quality Appraisal Using PEDro Scale.

PMVD, Point measures and variability data. Each item was scored either Yes, 1 or No, 0. Items 2-11 are summed for a PEDro total score. The sum of items 2-9 yields the internal validity subscale score, while the sum of items 10 and 11 yields the statistical reporting subscale score. The PEDro total score was rated 0-3, poor, 4-5 Moderate, 6-8 good, and 9-10= excellent.

### 3.2 Blood pressure assessment

Blood pressure assessment methods varied across the included studies. Most studies measured office blood pressure using standardized sphygmomanometry, whereas a smaller number employed ambulatory blood pressure monitoring or repeated clinical measurements. Variability in blood pressure measurement techniques may have contributed to the observed heterogeneity across studies and should be considered when interpreting the pooled findings.

### 3.3 Methodological quality of included studies

The methodological quality of the included studies was assessed using the PEDro Scale, which evaluates internal validity and statistical reporting of randomized controlled trials. The PEDro scale consists of 11 items, although the first item (eligibility criteria) is not included in the total score. The total score therefore ranges from 0 to 10, with higher scores indicating better methodological quality.

Across the 26 included studies, the mean PEDro score is 5.6 (ranging from 4 to 7), indicating moderate to good methodological quality overall. According to PEDro classification criteria, 0–3 indicates poor quality, 4–5 moderate quality, 6–8 good quality, and 9–10 excellent quality. Out of the 26 included trials, 16 studies were rated as moderate quality (PEDro score 4–5), 10 studies were rated as good quality (PEDro score 6–7; Table 5) and no study achieved excellent methodological quality. This distribution suggests that while most studies had acceptable methodological rigor, there remain methodological limitations that may influence the certainty of the pooled evidence.

#### 3.3.1 Randomization and allocation procedures

Most studies demonstrated adequate randomized allocation, which was reported in the majority of trials, including those by Ciolac (2008), Lamina (2010), Dimeo (2012), Venturelli (2015), Ash (2017), Boeno (2020), Punia (2020), Caminiti (2021), Javidi (2022), Qin (2022), and Pathan (2023). Randomization helps reduce selection bias and enhances comparability between intervention and control groups. However, allocation concealment was rarely reported across the included studies. Only one study, Sarinukul 2023 clearly reported concealed allocation. Lack of allocation concealment may increase the risk of selection bias, as investigators might influence group assignment.

#### 3.3.2 Baseline comparability

Many studies reported similar baseline characteristics between groups, which strengthens the internal validity of the trials. Baseline comparability was reported in studies such as Venturelli (2015), Boeno (2020), Punia (2020), Yakasai (2020), and Javidi (2022). This ensures that observed post-intervention differences in blood pressure outcomes are more likely attributable to the intervention rather than baseline group differences.

#### 3.3.3 Blinding

Blinding represents one of the most challenging methodological aspects in exercise intervention trials. Participant blinding was rarely implemented due to the nature of exercise training interventions. Therapist blinding was also largely absent. However, Assessor blinding was reported in a limited number of studies, including Venturelli (2015), Boeno (2020), Ngomane (2018), Javidi (2022), and Pathan (2023).

#### 3.3.4 Attrition and follow-up

Most studies adequately reported dropouts and follow-up rates, which strengthens confidence in the reported findings. Studies such as Ciolac (2008), Dimeo (2012), Venturelli (2015), Ash (2017), and Caminiti (2021) clearly reported participant attrition and follow-up outcomes. However, intention-to-treat analysis was seldom conducted.

#### 3.3.5 Statistical reporting

All included studies satisfied the statistical reporting criteria, as they provided between-group comparisons and measures of variability, such as standard deviations, confidence intervals, or standard errors. The availability of point measures and variability data (PMVD) allowed inclusion of these studies in the quantitative meta-analysis.

#### 3.3.6 Internal validity and reporting subscales

The internal validity subscale scores ranged from 2 to 5 out of 8, indicating moderate methodological rigor across most studies. The statistical reporting subscale scores were consistently 2 out of 2, reflecting adequate reporting of statistical outcomes.

### 3.4 Narrative synthesis

#### 3.4.1 Effects of exercise interventions on BP

##### 3.4.1.1 Aerobic exercise and BP

Aerobic exercise was the most commonly studied intervention. The majority of studies reported significant reductions in both SBP and DBP following aerobic exercise training. For example, Ciolac et al. (39) reported reductions of **−**7.1 mmHg in SBP and −10 mmHg in DBP following aerobic exercise intervention. Similarly, Lamina (40) demonstrated substantial reductions in SBP of **−**15.7 mmHg and −14.43 mmHg following continuous and interval training programs respectively. Other aerobic interventions such as stepping exercise, walking programs, and moderate-intensity endurance training Yakasai et al. (41), also demonstrated clinically meaningful reductions in BP, with SBP reductions ranging from −5 to −15 mmHg and DBP reductions ranging from −4 to −11 mmHg.

Endurance training among elderly hypertensive patients produced one of the largest reductions, with Venturelli et al. (42) reporting a −19.4 mmHg decrease in SBP and −8.0 mmHg decrease in DBP. Most aerobic exercise trials were rated as moderate methodological quality, although some, including Venturelli et al. (42) and Yakasai et al. (41), achieved good PEDro scores, strengthening the reliability of these findings. This indicates that there is strong evidence of aerobic training in reducing BP.

##### 3.4.1.2 Resistance training and blood pressure

Resistance training interventions included dynamic resistance exercise and isometric handgrip training. Isometric handgrip training showed notable antihypertensive effects in several studies. Javidi et al. (43) reported reductions of −15.5 mmHg in SBP and −5 mmHg in DBP, while Palmeira et al. (44) reported reductions of −8 mmHg in SBP and −4 mmHg in DBP. Similarly, Ash et al. (45) demonstrated modest reductions in SBP and DBP following resistance exercise interventions. However, findings were not entirely consistent. For example, Goncalves et al. (2014) reported a small decrease in SBP (−3.1 mmHg) but a slight increase in DBP (+1.0 mmHg). Resistance training studies generally demonstrated moderate to good methodological quality, with several trials e.g., Javidi et al., 2022 (43); Palmeira et al., 2021 (44) achieving good PEDro ratings. This indicates that there is conflicting evidence on the effects of strengthening exercise on BP.

##### 3.4.1.3 Combined exercise training and blood pressure

Combined aerobic and resistance training interventions also demonstrated significant improvements in blood pressure outcomes. For example, Alemayehu and Teferi (18) reported reductions of −12.5 mmHg in SBP and −9.75 mmHg in DBP following combined exercise training among hypertensive patients. Similarly, Ruangthai and Phoemsapthawee (46) reported reductions of −10.6 mmHg in SBP and −8.1 mmHg in DBP following combined training in elderly men. Caminiti (47) also reported reductions in both SBP and DBP following both aerobic and combined exercise training programs. These studies were generally rated as moderate methodological quality, although several demonstrated strong internal validities. Therefore, there is strong evidence on the effect of combined aerobic and resistance training in lowering BP.

##### 3.4.1.4 Secondary outcomes

Several studies reported improvements in secondary cardiovascular and functional outcomes, including:

1. Improved VO₂max and cardiorespiratory fitness
2. Reduced heart rate and blood pressure variability
3. Improved quality of life and physical performance
4. Favorable changes in body composition and inflammatory markers

These secondary benefits support the clinical relevance of exercise-based interventions beyond BP reduction.

### 3.5 Meta-analysis

#### 3.5.1 Effect of aerobic training on systolic blood pressure

The meta-analysis included 11 studies, comprising 302 participants in the aerobic training group and 308 participants in the control group. Across the studies, aerobic exercise consistently resulted in clinically meaningful reductions in SBP, typically ranging from −4.8 mmHg to −19.4 mmHg. Larger reductions were observed in studies involving moderate-to-vigorous intensity endurance training, interval training, and structured walking programs. The random-effects model meta-analysis demonstrated that aerobic training significantly reduced systolic blood pressure compared with control (95% CI −1.46 to −0.47; *p* = 0.0001). Substantial heterogeneity was observed across studies (I^2^ = 86%), indicating variability in intervention effects. (Figure 2a)

**Figure 2:**
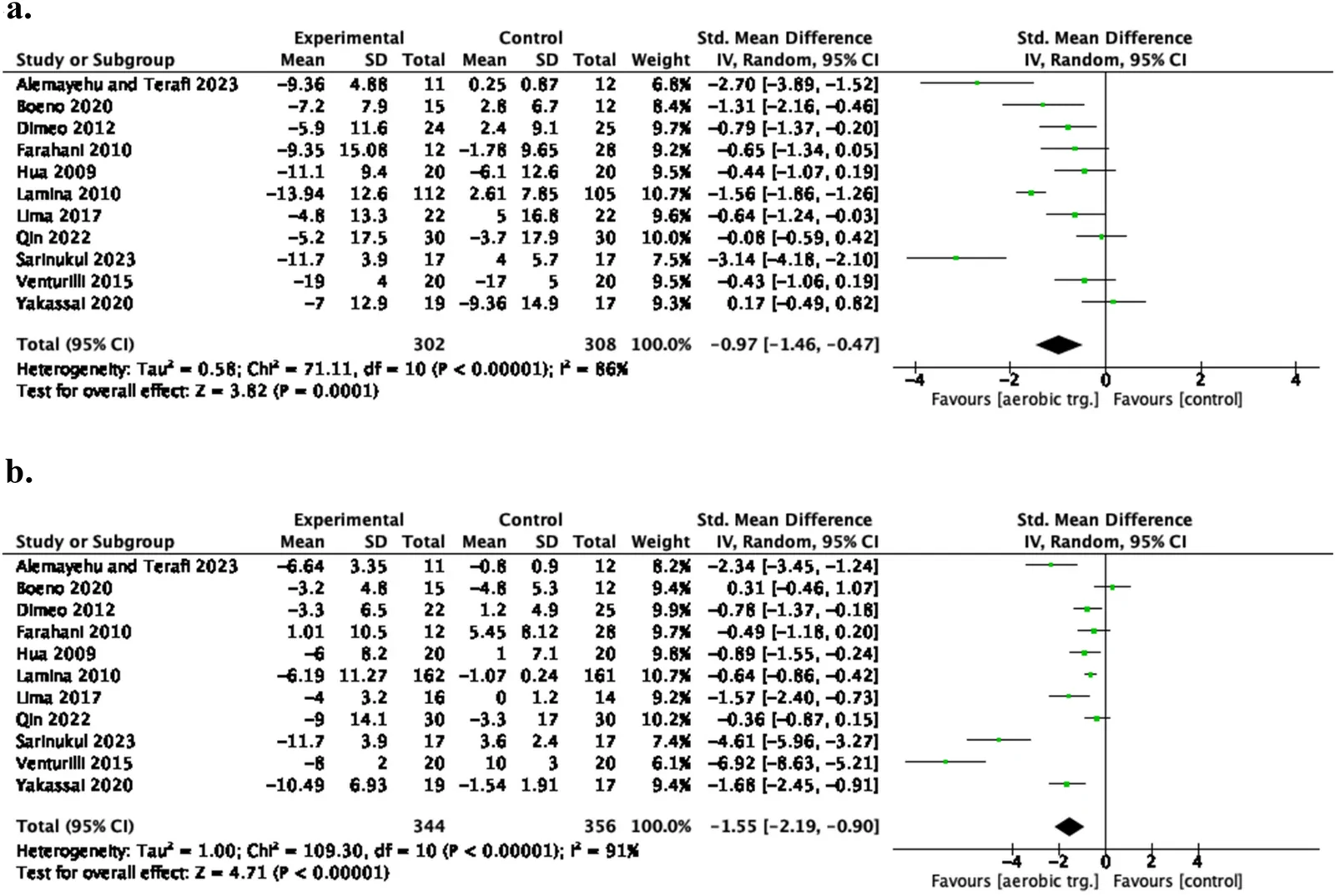
**(a)** Aerobic training vs. Systolic blood pressure for Hypertension. **(b)** Aerobic training vs. Diastolic blood pressure for Hypertension.

#### 3.5.2 Effect of aerobic training on diastolic blood pressure

The meta-analysis included 11 studies, comprising 344 participants in the aerobic training group and 356 participants in the control group. Aerobic exercise resulted in consistent and substantial reductions in DBP, typically between −3.0 mmHg and −11.7 mmHg. Larger effects were observed in endurance-based and moderate-intensity continuous training programs. The random-effects meta-analysis showed that aerobic training significantly reduced diastolic blood pressure compared with control conditions (SMD=−1.55; 95% CI −2.19 to −0.90; *p* < 0.00001). Substantial heterogeneity was observed across studies (I^2^ = 91%) (Figure 2b)

#### 3.5.3 Effect of resistance training on systolic blood pressure

The resistance-training meta-analysis included only two studies, comprising 26 participants in the intervention groups and 28 in the control groups. The pooled estimate did not demonstrate a statistically significant reduction in SBP compared with control (SMD=−1.13; 95% CI −3.43 to 1.17; *p* = 0.33), with substantial heterogeneity (I^2^ = 92%). Given the very small number of contributing studies and participants, this estimate should be interpreted cautiously and does not provide sufficient evidence to establish a reliable modality-specific effect of resistance training on SBP (Figure 3a).

**Figure 3:**
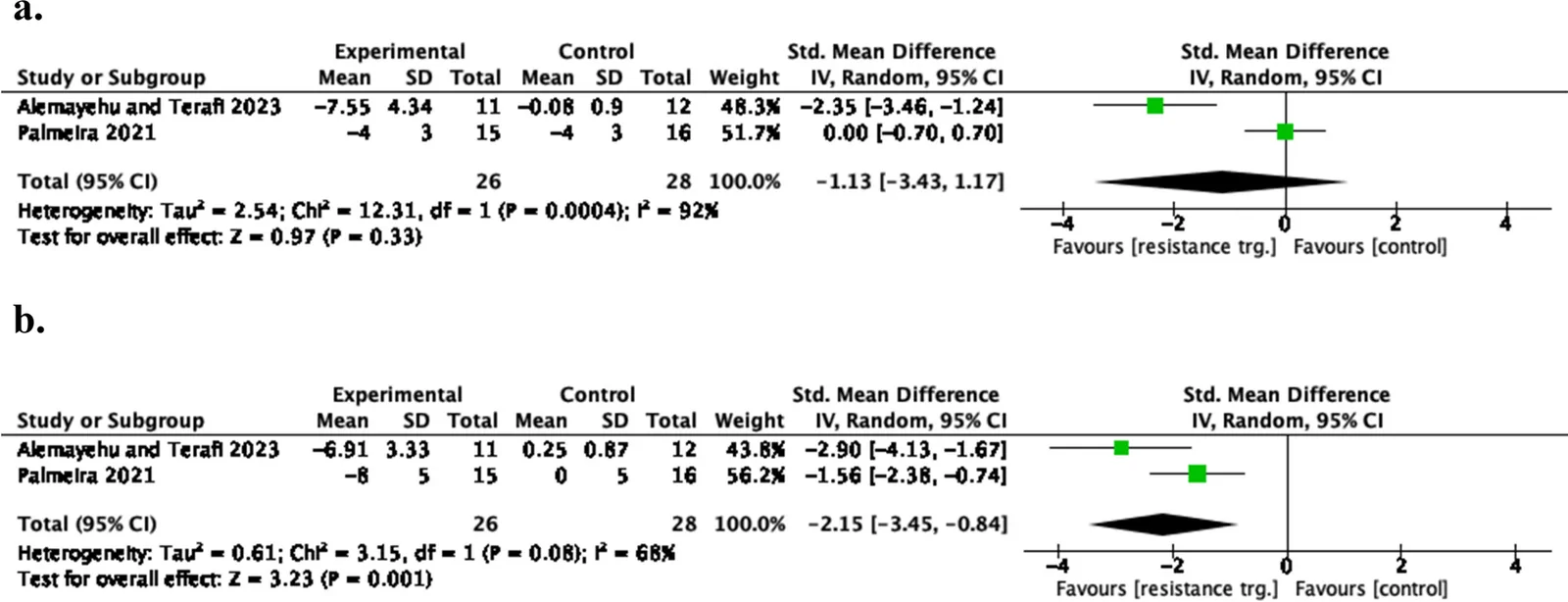
**(a)** Resistance training vs. Systolic blood pressure for Hypertension. **(b)** Resistance training vs. Diastolic blood pressure for Hypertension.

#### 3.5.4 Effect of resistance training on diastolic blood pressure

Two studies involving 26 intervention and 28 control participants contributed to the meta-analysis of DBP. The pooled estimate favoured resistance training (SMD=−2.15; 95% CI −3.45 to −0.84; *p* = 0.001; I^2^ = 68%). However, the estimate was derived from only two small studies, substantially limiting the precision and generalizability of the finding. Therefore, although a reduction in DBP was observed, the available evidence is insufficient to establish a reliable modality-specific effect of resistance training on DBP. Larger, adequately powered randomized controlled trials are required to confirm this finding (Figure 3b).

#### 3.5.5 Effect of combined training on systolic blood pressure

This meta-analysis included 4 studies, comprising 80 participants in the combined training group and 76 participants in the control group. Combined aerobic and resistance training generally produced significant SBP reductions, with reported decreases ranging from −4.1 mmHg to −12.5 mmHg. The random-effects meta-analysis showed that combined aerobic and resistance training significantly reduced systolic blood pressure compared with control (SMD=−1.11; 95% CI −2.11 to −0.11; *p* = 0.03). Substantial heterogeneity was observed across studies (I^2^ = 87%) (Figure 4a).

**Figure 4:**
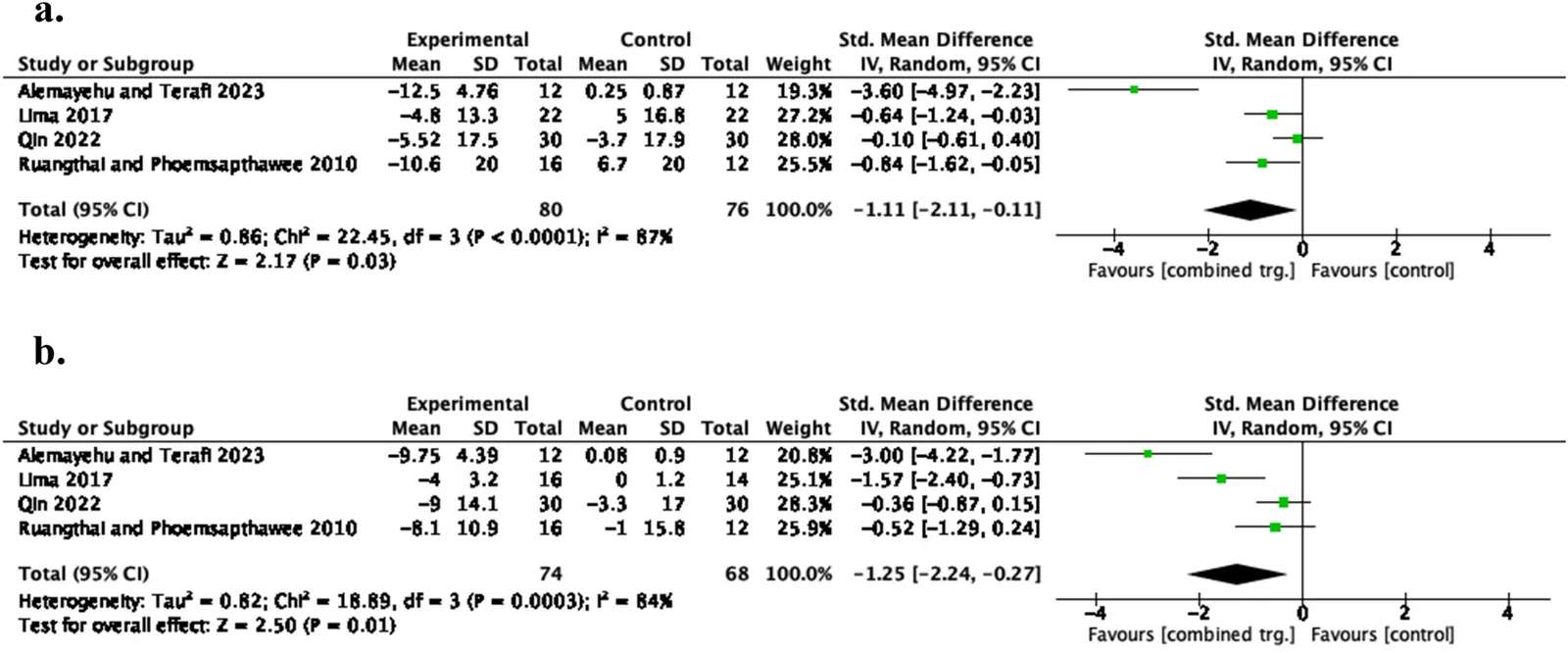
**(a)** Combined training vs. Systolic blood pressure for Hypertension. **(b)** Combine training vs. Diastolic blood pressure for Hypertension.

#### 3.5.6 Effect of combined training on diastolic blood pressure

This meta-analysis included 4 studies, with 74 participants in the combined training group and 68 participants in the control group. Combined training interventions resulted in significant reductions in DBP, with decreases ranging from −3.2 mmHg to −9.75 mmHg. These findings indicate that combined aerobic and resistance training produces a significant reduction in DBP favouring the intervention group. The random-effects meta-analysis demonstrated that combined aerobic and resistance training significantly reduced diastolic blood pressure compared with control (SMD=−1.25; 95% CI −2.24 to −0.27; *p* = 0.01). Substantial heterogeneity was observed across studies (I^2^ = 84%). (Figure 4b)

## 4 Discussion

This systematic review and meta-analysis evaluated the effects of different exercise training modalities on blood pressure outcomes in individuals with hypertension. The findings demonstrate that exercise training is an effective non-pharmacological intervention for BP reduction, with aerobic and combined aerobic–resistance training showing the most consistent and clinically meaningful reductions in both SBP and DBP. In contrast, resistance training alone showed a clear benefit for DBP but produced inconsistent and non-significant effects on SBP. The present findings should primarily be interpreted within the context of hypertension management because majority of included studies enrolled participants with established hypertension. Although a small number of studies included individuals with prehypertension, these represented only a minor proportion of the available evidence and are unlikely to have substantially influenced the overall conclusions.

The most robust evidence emerged for aerobic training, which consistently reduced SBP and DBP across diverse populations, intervention durations, and exercise formats. Epidemiological evidence indicates that a sustained 5 mmHg reduction in systolic blood pressure may reduce stroke mortality by approximately 14%, coronary heart disease mortality by 9%, and all-cause mortality by approximately 7%. Therefore, the reductions observed in this review are clinically meaningful. These findings reinforce current international guidelines that recommend aerobic exercise as the cornerstone of lifestyle management for hypertension (8, 28, 29).

Combined aerobic and resistance training also demonstrated significant reductions in both SBP and DBP. The pooled effects suggest that combining exercise modalities may provide additive or complementary physiological benefits, potentially through simultaneous improvements in vascular function, autonomic regulation, and muscular fitness. However, the presence of substantial heterogeneity indicates that the effectiveness of combined training may depend on how the aerobic and resistance components are prescribed and integrated. The findings concerning resistance training require substantially greater caution. Although the pooled analysis showed a statistically significant reduction in DBP, this estimate was based on only two studies involving 54 participants in total and was accompanied by moderate heterogeneity This differential response may reflect the distinct physiological mechanisms underlying SBP and DBP regulation. Resistance training may preferentially influence peripheral vascular resistance and autonomic balance, which are more closely linked to DBP, whereas SBP appears to be more responsive to improvements in arterial compliance and cardiorespiratory fitness typically induced by aerobic exercise.

The substantial heterogeneity observed across the pooled analyses (I^2^ = 68%–92%) is likely multifactorial. Considerable differences existed in participant characteristics, including age, baseline BP, comorbidities, antihypertensive medication use, and physical fitness. Exercise interventions also varied substantially with respect to modality (aerobic, resistance or combined training), intensity, frequency, duration, supervision and adherence. Furthermore, differences in BP assessment methods, intervention duration, sample sizes and methodological quality may have contributed to the observed variability. Although these factors reduce the precision of the pooled estimates, the direction of treatment effect was generally consistent across studies, with most trials demonstrating reductions in systolic and/or diastolic BP. Therefore, the findings support the beneficial role of exercise in hypertension management while highlighting the need for more standardized intervention protocols and adequately powered randomized trials.

The findings of the present review are broadly consistent with the established evidence that structured exercise reduces blood pressure among adults with elevated blood pressure and hypertension. However, the magnitude and comparative effectiveness of individual exercise modalities remain less certain. Pescatello et al. (33) reported broad evidence supporting physical activity for BP reduction across normal, prehypertensive and hypertensive populations, whereas Edwards et al. (34), in a large network meta-analysis of 270 randomized controlled trials involving 15,827 participants, demonstrated significant reductions in resting SBP and DBP across aerobic, dynamic resistance, combined and isometric exercise modalities. The present review adds a more focused synthesis of contemporary randomized evidence identified through 2024 and separates the findings according to aerobic, resistance and combined training. The aerobic and combined-training analyses demonstrated statistically significant reductions in both SBP and DBP, although substantial heterogeneity was present. These findings are therefore best interpreted as confirming the robustness of exercise as a non-pharmacological strategy while illustrating variability according to exercise prescription and study characteristics. Importantly, the resistance-training evidence in the present review was substantially less extensive than that available in larger modality-specific reviews, and the pooled resistance estimate was based on only two studies. Consequently, the resistance-training findings should be considered preliminary and hypothesis-generating rather than definitive. The findings have important clinical implications. The present findings suggest that aerobic exercise may be considered the preferred exercise modality for blood pressure management because it demonstrated the most consistent evidence across studies. Nevertheless, exercise prescriptions should remain individualized according to patient characteristics, comorbidities, functional capacity and personal preferences. Combined exercise may offer additional benefits where feasible, whereas resistance training appears to serve as a useful adjunct rather than a replacement for aerobic exercise.

A major strength of this review is the comprehensive synthesis of multiple exercise modalities and the inclusion of both meta-analytic and narrative evidence. However, some limitations still exist including; A small number of included studies enrolled participants with prehypertension, although most participants had established hypertension. Therefore, the findings are primarily generalizable to adults with hypertension. Furthermore, variation in blood pressure measurement methods across studies may have contributed to the substantial heterogeneity observed in the pooled analyses. Many included studies had small sample sizes, short intervention durations, and varying methodological quality. The substantial heterogeneity limits the certainty of pooled estimates, and publication bias could not be excluded. Additionally, long-term adherence and sustainability of exercise interventions were rarely reported. Future studies should focus on head-to-head comparisons of exercise modalities, standardized intervention protocols, and longer follow-up periods to assess sustained blood pressure control. Studies conducted in low- and middle-income countries are particularly needed to enhance the global applicability of findings

## 5 Conclusion

The available evidence suggests that aerobic exercise, either alone or combined with resistance training, appears to be associated with clinically meaningful reductions in systolic and diastolic BP among adults with hypertension**.** However, the certainty of these findings is moderated by substantial between-study heterogeneity and the moderate methodological quality of many included trials. Therefore, although exercise should remain an important component of hypertension management, additional high-quality randomized controlled trials using standardized exercise protocols are needed to strengthen the evidence base.

## References

[1] Xu X Islam SMS Schlaich M Jennings G Schutte AE. The contribution of raised blood pressure to all-cause and cardiovascular deaths and disability-adjusted life-years (DALYs) in Australia: analysis of global burden of disease study from 1990 to 2019. PLoS One. (2024) 19(2):e0297229. 10.1371/journal.pone.0297229 38381709 PMC10881002

[2] Chobanian AV Bakris GL Black HR Cushman WC Green LA Izzo JL. Seventh report of the joint national committee on prevention, detection, evaluation, and treatment of high blood pressure. Hypertension. (2003) 42(6):1206–52. 10.1161/01.HYP.0000107251.49515.c2 14656957

[3] World Health Organization. A global brief on hypertension: silent killer, global public health crisis: World Health Day 2013. World Health Organization. (2013). 201WHO/DCO/WHD/2013.2. Available online at: https://iris.who.int/handle/10665/79059 (Accessed January 22, 2026).

[4] Cohen DL Townsend RR Angell SY DiPette DJ. The world health organization recognizes noncommunicable diseases and raised blood pressure as global health priority for 2025. J Clin Hypertens Greenwich Conn. (2014) 16(9):624. 10.1111/jch.12384 PMC5723123 25087601

[5] Lu W- Yuan J- Liu Z- Su Z-H Shen Y-C Li S- Worldwide trends in mortality for hypertensive heart disease from 1990 to 2019 with projection to 2034: data from the global burden of disease 2019 study. Eur J Prev Cardiol. (2024) 31(1):23–37. 10.1093/eurjpc/zwad262 37665956

[6] Organization WH Global report on hypertension: the race against a silent killer. WHO. (2023) https://books.google.com/books?hl=en&lr=&id=KaIOEQAAQBAJ&oi=fnd&pg=PR5&dq=who+incidence+of+hypertension&ots=AlcwmAHePE&sig=ZmVgAKO7SizgzThb5IImF2mZLIs (Accessed January 22, 2026).

[7] Booth JN 3rd Li J Zhang L Chen L Muntner P Egan B. Trends in prehypertension and hypertension risk factors in US adults: 1999-2012. Hypertens Dallas Tex (1979) 70(2):275–84. 10.1161/HYPERTENSIONAHA.116.09004 PMC5594566 28607131

[8] Miller CS Glick M Rhodus NL. Hypertension guidelines: new opportunities and challenges. J Am Dent Assoc. (2017) 149(4):229–31. 10.1016/j.adaj.2018.01.047 29599014

[9] Anota A Nedi T. Blood pressure control and associated factors among hypertensive patients attending shashemene referral hospital, oromia, Ethiopia. Hosp Pharm. (2022) 57(4):555–63. 10.1177/00185787211061372 35898241 PMC9310308

[10] Ahmadi S Sajjadi H N Nejad F Ahmadi N Karimi SE Yoosefi M. Lifestyle modification strategies for controlling hypertension: how are these strategies recommended by physicians in Iran? Med J Islam Repub Iran. (2019) 33: 43. 10.34171/mjiri.33.43 31456967 PMC6708116

[11] Alpsoy Ş Exercise and hypertension. In: Xiao J, editor. Physical Exercise for Human Health. Singapore: Springer Singapore (2020) 1228 153–67. (Advances in Experimental Medicine and Biology). 10.1007/978-981-15-1792-1_10 32342456

[12] Aguilar-Cordero MJ Rodríguez-Blanque R Leon-Ríos X Expósito Ruiz M García García I Sánchez-López AM. Influence of physical activity on blood pressure in children with overweight/obesity: a randomized clinical trial. Am J Hypertens. (2020) 33(2):131–6. 10.1093/ajh/hpz174 31678988

[13] AHC MEDIA. Blood pressure control: exercise vs. Meds. Intern Med Alert. (2019) 41(16).

[14] Ahn N Kim K. Can active aerobic exercise reduce the risk of cardiovascular disease in prehypertensive elderly women by improving HDL cholesterol and inflammatory markers? Int J Environ Res Public Health. (2020) 17:5910 17(16). 10.3390/ijerph17165910 32824020 PMC7459903

[15] Bakker EA Sui X Brellenthin AG Lee D-C. Physical activity and fitness for the prevention of hypertension. Curr Opin Cardiol. (2018) 33(4):394–401. 10.1097/HCO.0000000000000526 29762150

[16] Ahmad AM Mahmoud AM Serry ZH Mohamed MM Elghaffar HAA. Effects of low-versus high-volume high-intensity interval training on glycemic control and quality of life in obese women with type 2 diabetes. A randomized controlled trial. J Exerc Sci Fit. (2023) 21(4):395–404. 10.1016/j.jesf.2023.08.003 37954548 PMC10632101

[17] Abrahin O Abrahin RP Guimarães M De Holanda VBT Figueiredo FADPL Viana Rosa B. Blood pressure responsiveness to resistance training in the hypertensive older adult: a randomized controlled study. Blood Press Monit. (2024) 29(2):71–81. 10.1097/MBP.0000000000000690 38300019

[18] Alemayehu A Teferi G. Effectiveness of aerobic, resistance, and combined training for hypertensive patients: a randomized controlled trial. Ethiop J Health Sci. (2023) 33(6):1063–74. 10.4314/ejhs.v33i6.17 38784482 PMC11111269

[19] Kodela P Okeke M Guntuku S Lingamsetty SSP Slonovschi E. Management of hypertension with non-pharmacological interventions: a narrative review. Cureus. (2023) 15(8):e43022. 10.7759/cureus.43022 37674940 PMC10478605

[20] Ballut OM Alzahrani AA Alzahrani RA Alzahrani AT Alzahrani RA Alzahrani MF. The impact of non-pharmacological interventions on blood pressure control in patients with hypertension: a systematic review. Cureus. (2023) 15(11):e48444. 10.7759/cureus.48444 38074046 PMC10702403

[21] Mobasseri M Yavari A Najafipoor F Aliasgarzadeh A Niafar M. Effect of a long-term regular physical activity on hypertension and body mass index in type 2 diabetes patients. J Sports Med Phys Fitness. (2015) 55(1–2):1–2. 24947808

[22] Kathia MM Bommarito JC Hinks A Leake E Shannon J Pitman J. Physiological and clinical responses to cycling 7850 Km over 85 Days in a physically active middle-aged man with idiopathic Parkinson's Disease. Physiol Rep. (2023) 11(14):e15772. 10.14814/phy2.15772 37474301 PMC10359154

[23] Chehuen M Andrade-Lima A Silva N Miyasato R Souza RWA Leicht A. Physiological responses to maximal and submaximal walking in patients with symptomatic peripheral artery disease. Arq Bras Cardiol. (2021) 117(2):309–16. 10.36660/abc.20200156 34495225 PMC8395785

[24] Croymans DM Krell SL Oh CS Katiraie M Lam CY Harris RA. Effects of resistance training on central blood pressure in obese young men. J Hum Hypertens. (2014) 28(3):157–64. 10.1038/jhh.2013.81 24005959 PMC4119468

[25] Brown Wright L Gregoski MJ Tingen MS Barnes VA Treiber FA. Impact of stress reduction interventions on hostility and ambulatory systolic blood pressure in African American adolescents. J Black Psychol. (2011) 37(2):210–33. 10.1177/0095798410380203 22485058 PMC3319013

[26] Campbell NR Burnens MP Whelton PK Angell SY Jaffe MG Cohn J. 2021 World health organization guideline on pharmacological treatment of hypertension: policy implications for the region of the americas. Lancet Reg Heal. (2022) 9. Available online at: https://www.thelancet.com/journals/lanam/article/PIIS2667-193X(22)00036-9/fulltext (Accessed January 22, 2026). 10.1016/j.lana.2022.100219 PMC9107389 35711684

[27] DePalma SM Himmelfarb C MacLaughlin EJ Taler SJ. Hypertension guideline update: a new guideline for a new era. JAAPA J Am Acad Physician Assist Lippincott Williams Wilkins. (2018) 31(6):16–22. 10.1097/01.JAA.0000533656.93911.38 29762202

[28] Ding D Mutrie N Bauman A Pratt M Hallal PR Powell KE. Physical activity guidelines 2020: comprehensive and inclusive recommendations to activate populations. Lancet. (2020) 396(10265):1780–2. 10.1016/S0140-6736(20)32229-7 33248019

[29] Cornelissen VA Verheyden B Aubert AE Fagard RH. Effects of aerobic training intensity on resting, exercise and post-exercise blood pressure, heart rate and heart-rate variability. J Hum Hypertens. (2010) 24(3):3–182. 10.1038/jhh.2009.51 19554028

[30] Angraini R Irwan AM Arafat R. Effectiveness of hydrotherapy to lower blood pressure in hypertensive patients: a systematic review. Int J Caring Sci. (2023) 16(3):1237–53.

[31] Baffour-Awuah B Pearson MJ Dieberg G Smart NA. Isometric resistance training to manage hypertension: systematic review and meta-analysis. Curr Hypertens Rep. (2023) 25(4):35–49. 10.1007/s11906-023-01232-w 36853479 PMC10014822

[32] Baffour-Awuah B Dieberg G Pearson MJ Smart NA. Blood pressure control in older adults with hypertension: a systematic review with meta-analysis and meta-regression. Int J Cardiol Hypertens. (2020) 6: 100040. 10.1016/j.ijchy.2020.100040 33447766 PMC7803055

[33] Pescatello LS Buchner DM Jakicic JM Powell KE Kraus WE Bloodgood B. Physical activity to prevent and treat hypertension: a systematic review. Med Sci Sports Exerc. (2019) 51(6):1314–23. 10.1249/MSS.0000000000001943 31095088

[34] Edwards JJ Deenmamode AHP Griffiths M Arnold O Cooper NJ Wiles JD. Exercise training and resting blood pressure: a large-scale pairwise and network meta-analysis of randomised controlled trials. Br J Sports Med. (2023) 57(20):1317–26. 10.1136/bjsports-2022-106503 37491419

[35] Page MJ McKenzie JE Bossuyt PM Boutron I Hoffmann TC Mulrow CD. The PRISMA 2020 statement: an updated guideline for reporting systematic reviews. Br Med J. (2021) n71:n71. 10.1136/bmj.n71 PMC8005924 33782057

[36] Ouzzani M Hammady H Fedorowicz Z Elmagarmid A. Rayyan—a web and mobile app for systematic reviews. Syst Rev. (2016) 5(1):210. 10.1186/s13643-016-0384-4 27919275 PMC5139140

[37] Mukhtar NB Meeus M Gursen C Mohammed J D Pauw R Cagnie B. Effectiveness of hands-off therapy in the management of primary headache: a systematic review and meta-analysis. Eval Health Prof. (2022) 45(2):183–203. 10.1177/0163278720983408 33406891

[38] Bakker EW Verhagen AP Van Trijffel E Lucas C Koes BW. Spinal mechanical load as a risk factor for low back pain: a systematic review of prospective cohort studies. Spine. (2009) 34(8):E281–93. 10.1097/BRS.0b013e318195b257 19365237

[39] Ciolac EG Guimarães GV D'Avila VM Bortolotto LA Doria EL Bocchi EA. Acute aerobic exercise reduces 24-h ambulatory blood pressure levels in long-term-treated hypertensive patients. Clin Sao Paulo Braz. (2008) 63(6):753–8. 10.1590/s1807-59322008000600008 PMC2664274 19060996

[40] Lamina S. Effects of continuous and interval training programs in the management of hypertension: a randomized controlled trial. J Clin Hypertens Greenwich Conn. (2010) 12(11):841–9. 10.1111/j.1751-7176.2010.00315.x PMC8816431 21054770

[41] Yakasai AM Maharaj SS Nuhu JM Danazumi MS. Moderate intensity endurance exercise: a beneficial intervention for relative cardiovascular parameters of primary and secondary hypertensive patients. Randomised Controlled Trial. Eur J Physiother. (2021) 23(4):259–65. 10.1080/21679169.2020.1720800

[42] Venturelli M Cè E Limonta E Schena F Caimi B Carugo S. Effects of endurance, circuit, and relaxing training on cardiovascular risk factors in hypertensive elderly patients. Age Dordr Neth. (2015) 37(5):101. 10.1007/s11357-015-9835-4 PMC5005840 26381921

[43] Javidi M Ahmadizad S Argani H Najafi A Ebrahim K Salehi N. Effect of lower- versus higher-intensity isometric handgrip training in adults with hypertension: a randomized controlled trial. J Cardiovasc Dev Dis. (2022) 9(9):287. 10.3390/jcdd9090287 36135432 PMC9500826

[44] Palmeira AC Farah BQ Silva GO Moreira SR Barros MVG Correia MA. Effects of isometric handgrip training on blood pressure among hypertensive patients seen within public primary healthcare: a randomized controlled trial. Sao Paulo Med J Rev Paul Med. (2021) 139(6):648–56. 10.1590/1516-3180.2020.0796.R1.22042021 PMC9634841 34787298

[45] Ash GI Taylor BA Thompson PD MacDonald HV Lamberti L Chen M-H. The antihypertensive effects of aerobic versus isometric handgrip resistance exercise. J Hypertens. (2017) 35(2):291–9. 10.1097/HJH.0000000000001176 27861249 PMC5713890

[46] Ruangthai R Phoemsapthawee J Makaje N Phimphaphorn P. Comparative effects of water- and land-based combined exercise training in hypertensive older adults. Arch Gerontol Geriatr. (2020) 90:104164. 10.1016/j.archger.2020.104164 32650155

[47] Caminiti G Iellamo F Mancuso A Cerrito A Montano M Manzi V. Effects of 12 weeks of aerobic versus combined aerobic plus resistance exercise training on short-term blood pressure variability in patients with hypertension. J Appl Physiol. (2021) 130(4):1085–92. 10.1152/japplphysiol.00910.2020 33630677

